# SUPPORTING DIABETES MANAGEMENT FOR PERSONS LIVING WITH DEMENTIA: CARE PARTNER EXPERIENCES

**DOI:** 10.1093/geroni/igae098.0635

**Published:** 2024-12-31

**Authors:** Alycia Bristol, Synneva Hagen-Lillevik, Shinduk Lee, Nancy Allen

**Affiliations:** University of Utah, Salt Lake City, Utah, United States; University of Utah, Salt Lake City, Utah, United States; University of Utah, Salt Lake City, Utah, United States; University of Utah, Salt Lake City, Utah, United States

## Abstract

Care partners (CP) play a vital role in assisting persons living with dementia (PLWD) but often face challenges when supporting type 2 diabetes (T2D) management. We conducted pilot work to explore adapting an existing diabetes self-management education/support intervention called Share plus for CP of PLWD with T2D. Share plus promotes continuous glucose monitoring with CP data sharing and provides dyadic communication, problem-solving, and action planning strategies. Two focus groups (n=5, n=4) were conducted with CP. Focus groups were recorded, transcribed, and analyzed using content analysis. CP also completed surveys, including the Partner Diabetes Distress Scale (PDDS), the Zarit Burden Scale, and one burden question specific to diabetes. Descriptive statistics were used for analysis. As part of the larger pilot work, we found that CP expressed frustration with diabetes management for PLWD and discussed their lack of knowledge of addressing the impact of dementia symptoms on diabetes management. Across three PDDS questions, CP reported varying frustration levels ranging from none (44.4%) to a lot (11.1%). Additionally, CP reported experiencing slight (44.4%) and moderate (22.2%) burden when supporting diabetes management. During focus groups, CP identified frustrations and struggles in managing dementia symptoms’ influence on diabetes management, particularly regarding diet, medications, and communication. Additionally, CP reported uncertainty about how to support PLWD’s need for independence. The next steps are to develop strategies to tailor the Share plus intervention by incorporating the unique challenges faced by PLWD and their CP, such as creating specific diabetes and dementia communication strategies for CP.

